# From linguistic imperialism to algorithmic dominance: psychological implications of language hegemony in the age of digital intelligence

**DOI:** 10.3389/fpsyg.2026.1853125

**Published:** 2026-05-20

**Authors:** Jianbu Yang, Jie Zeng, Xingpeng Zheng

**Affiliations:** 1 School of Culture and Education, Tianfu College of SWUFE, Chengdu, China; 2 School of Foreign Languages, Chengdu Normal University, Chengdu, China; 3 School of Management, Chongqing Institute of Engineering, Chongqing, China

**Keywords:** algorithmic dominance, communicative confidence, digital intelligence, language anxiety, language hegemony, willingness to communicate

## Abstract

This article examines how language hegemony is being reconfigured in the age of digital intelligence and its psychological implications for language users, particularly regarding confidence, anxiety, and willingness to communicate. While research on linguistic imperialism has shown how dominant languages consolidate power through history, institutions, and ideology, less attention has been paid to how digital infrastructures reproduce linguistic inequality and shape speakers’ confidence, perceived legitimacy, and communicative behaviour. The article argues that language hegemony increasingly operates through algorithmic dominance, reinforced by unequal data representation, platform visibility, uneven AI performance, and the naturalisation of dominant languages as default media of global communication. It proposes a four-dimensional framework of data dominance, platform dominance, model dominance, and ideological dominance. This framework suggests that language inequality in the age of digital intelligence is not only structurally reproduced but also psychologically consequential, influencing language anxiety, communicative confidence, willingness to communicate, and perceptions of linguistic adequacy. The article thus extends debates on linguistic imperialism into the era of platform governance and generative AI, while calling for greater attention to multilingual justice, digital inequality, the psychology of language technology, and speakers’ strategies of adaptation and resistance.

## 1 Introduction

Language is not only a means of communication. It is also tied to authority, legitimacy, and social value. This insight runs through critical work on language, from Gramsci’s notion of hegemony to Bourdieu’s account of symbolic power (Gramsci, 1971; Bourdieu, 1991). From this perspective, the prominence of some languages over others cannot be explained simply by wider circulation or practical utility. It is bound up with histories of empire, schooling, mobility, publishing, and labour markets, as well as with institutions that render some linguistic forms more legitimate than others (Phillipson, 1992, 2009, 2022; Pennycook, 1994). The concept of linguistic imperialism remains useful because it shows that language inequality is organised rather than accidental. Later work on native-speakerism and raciolinguistic ideology sharpened this point by showing how domination often persists through ordinary judgements about correctness, fluency, accent, and worth (Holliday, 2006; Flores and Rosa, 2015). Such judgements may shape whether speakers experience their own linguistic resources as legitimate and whether participation feels possible or risky.

Such questions arise differently in the age of digital intelligence, understood here as a sociotechnical environment shaped by platforms, algorithmic systems, and AI-mediated communication. Language now circulates through platforms, search systems, machine translation, speech recognition, and generative AI. These are not neutral channels; they sort, rank, and amplify language in ways that matter socially and psychologically. Recent studies suggest that languages do not enter such systems on equal terms. Technological support remains uneven, favouring some dominant languages through richer digital resources, stronger tools, and greater visibility (Grützner-Zahn et al., 2024). Zaugg et al. (2022) describe many less-supported languages as digitally disadvantaged, while UNESCO (2025) argues that meaningful multilingual participation increasingly depends on whether languages can be represented and processed in digital environments. If a language is less visible, less searchable, or less usable online, this may shape speakers’ confidence, perceived legitimacy, and willingness to participate.

Building on Zeng et al.’s (2023) discussion of English linguistic neo-imperialism, this article argues that language hegemony is increasingly reproduced through algorithmic dominance. By this, we mean the uneven advantages that certain languages acquire through data concentration, platform visibility, model performance, and the assumption that what works better technically must be better in itself. This perspective article offers a conceptual account of how digital systems are reworking language inequality and why that process matters not only for linguistic justice but also for language users’ self-perception, communicative confidence, language anxiety, and willingness to communicate. These constructs draw on established work on communicative confidence, foreign language anxiety, and willingness to communicate in language psychology and second language acquisition (SLA) research (Clément, 1980; Horwitz et al., 1986; MacIntyre et al., 1998). As a perspective article, it does not empirically test hypotheses; rather, it develops a conceptual framework that may guide future research on the psychological consequences of digitally mediated language inequality.

## 2 From linguistic imperialism to algorithmic dominance

The concept of linguistic imperialism is still difficult to dispense with because it reminds us that language inequality is never just about language. Phillipson (1992) used the term to show how English came to occupy a privileged position through structural and cultural asymmetries, especially in education, administration, and knowledge production. His point was not simply that English spread, but that it did so within an unequal order that made it appear more valuable, more legitimate, and more consequential than other languages. Later work showed that this order did not disappear with the retreat of formal colonial power. If anything, it became harder to grasp because it was reproduced less through direct imposition than through institutions, markets, and ordinary forms of consent (Phillipson, 2009, 2022). In that sense, linguistic imperialism names more than a historical episode. It points to a durable structure in which one language comes to stand for mobility, competence, authority, and access, while others are pushed towards the margins.

At the same time, the present moment calls for a further shift in emphasis. Recent scholarship has shown that the contemporary dominance of English is often reproduced through aspiration as much as regulation, and through routine practice as much as external pressure. In both postcolonial and non-colonial settings, English is frequently sustained because it is linked to education, employability, cosmopolitanism, and symbolic advancement (Lai, 2021; Tupas, 2022). Particularly useful here is Zeng et al.’s (2023) discussion of linguistic neo-imperialism, which captures the more diffuse character of language domination under globalisation. Their argument is persuasive precisely because it moves away from a narrowly top-down picture and shows how hierarchy is maintained in everyday life—through communication, business, academia, and education—by subtle, locally reproduced mechanisms that are often taken for granted (Zeng et al., 2023). Even so, that reformulation still speaks mainly to institutional and ideological processes. It does not fully address the infrastructures through which language is now increasingly encountered, sorted, and acted upon.

This is where algorithmic dominance becomes useful. The term is meant to capture more than the simple fact that language now passes through digital systems. The issue is that such systems do not process all languages equally. They privilege some, neglect others, and in doing so reshape older language hierarchies in new ways. In the age of digital intelligence, power is exercised not only through policy, schooling, and cultural prestige but also through data accumulation, platform visibility, model performance, and infrastructural design. Some languages are better represented in corpora, easier to search, more reliably translated, and more effectively generated in AI-mediated environments, while others remain sparse, unstable, or weakly supported. Work in multilingual natural language processing (NLP) has repeatedly shown that high-resource languages receive disproportionate attention in data collection, evaluation, and model development, whereas low-resource languages continue to face systematic underrepresentation (Joshi et al., 2020; Grützner-Zahn et al., 2024). That imbalance is often treated as merely technical, but it is not. It is one way language inequality is being reconstituted under computational conditions.

To speak of algorithmic dominance, then, is not to suggest that linguistic imperialism has somehow become irrelevant. Rather than replacing earlier frameworks, algorithmic dominance extends them into new technological conditions. Linguistic imperialism helps explain the historical making of unequal language orders; algorithmic dominance helps explain how those orders are now reproduced through infrastructures that appear neutral, efficient, and politically innocent. This point also echoes work in the philosophy of technology, which shows that technical systems can embody social and political arrangements rather than merely serve as neutral tools (Winner, 2007). This matters because the mode of reproduction has changed. Language hierarchy is no longer maintained only through school systems, publishing markets, state policy, or elite aspiration. It is also built into recommender systems, machine translation pipelines, speech technologies, search architectures, and generative AI models. Under such conditions, dominant languages do not simply remain socially powerful. They become easier to compute, easier to circulate, and easier to accept as normal. That shift has consequences not only for language distribution in society but also for how speakers evaluate their own linguistic adequacy, imagine their communicative possibilities, and position themselves within digitally mediated interaction.

Seen in this light, the move from linguistic imperialism to algorithmic dominance is best understood as an attempt to update, rather than discard, earlier critical theory. It allows us to hold on to the insight that language inequality is historically produced, while recognising that it is now increasingly reproduced through computational infrastructures and lived as a matter of confidence, legitimacy, and communicative possibility. The next section develops this argument through four related dimensions: data dominance, platform dominance, model dominance, and ideological dominance. These dimensions are used here as conceptual categories rather than empirically tested variables, helping to explain how older language hierarchies may be translated into new forms of technological and psychological inequality.

## 3 Discussion

### 3.1 A framework of algorithmic dominance in language hegemony

**Figure 1:**
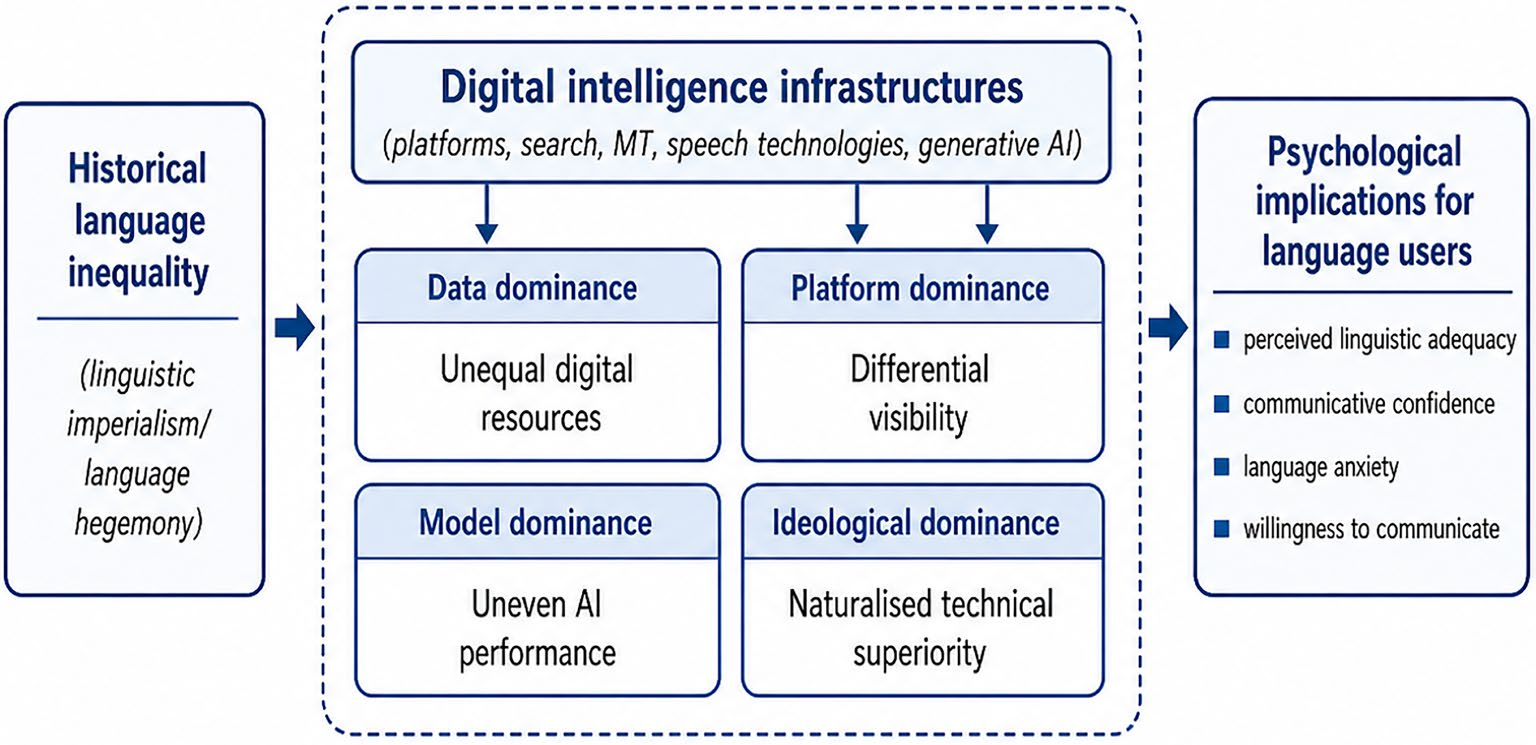
A framework of algorithmic dominance and its psychological implications.

If linguistic imperialism helps explain the historical and ideological conditions under which dominant languages acquired authority, algorithmic dominance directs attention to the infrastructures through which that authority is now renewed. The point is not that digital technologies created linguistic hierarchy from scratch. What has changed is the mode through which older asymmetries are reproduced. In the age of digital intelligence, language hierarchy is increasingly built into systems that collect data, allocate visibility, optimise performance, and normalise some languages as more processable, usable, and central than others (Joshi et al., 2020; Grützner-Zahn et al., 2024). This shift cannot be reduced to a technical rearrangement. It also affects how speakers imagine their own linguistic worth and communicative possibilities. As shown in Figure 1, algorithmic dominance can be understood through four interrelated dimensions.

To bring these issues into sharper view, we propose a four-part framework: data dominance, platform dominance, model dominance, and ideological dominance. These dimensions are interconnected, but each highlights a different route through which language hegemony acquires psychological force. This framework shares some concerns with and extends Zaugg et al.’s (2022) descriptive account of digitally disadvantaged languages by treating digital disadvantage as algorithmic dominance, that is, as a power relation linking infrastructural asymmetry to ideological naturalisation and psychological internalisation. Data dominance concerns the unequal distribution of digital language resources and how this shapes what languages can be learned and supported computationally (Bender, 2019; Joshi et al., 2020). Platform dominance concerns circulation and visibility, namely, which languages are easy to find, amplify, and encounter, and which remain peripheral in digitally mediated interaction (Zaugg et al., 2022; UNESCO, 2025). Model dominance refers to uneven AI performance across languages, with consequences for usability, confidence, and dependence in everyday communication (Grützner-Zahn et al., 2024; McGiff and Nikolov, 2025). Ideological dominance captures the tendency for such asymmetries to be normalised, so that historically produced inequalities begin to appear as common-sense facts about efficiency, universality, or technical superiority (Phillipson, 2022; Zeng et al., 2023).

The value of this framework lies in connecting structural inequality with lived psychological consequences. For language users, these conditions matter because they shape whether one’s language feels supported, whether one’s repertoire seems legitimate, and whether participation appears inviting or risky. They also shape how speakers evaluate their own linguistic adequacy and anticipate judgement in digitally mediated interaction. Psychologically, such effects are likely to emerge through repeated user experience: lower visibility, weaker technological support, or poorer AI performance may be interpreted not only as system limitations, but also as signs that one’s language is less adequate for digitally mediated communication. Over time, these repeated encounters may affect perceived legitimacy, communicative self-confidence, language anxiety, and willingness to communicate. The mechanism proposed here is therefore not direct technological determinism, but a process of exposure, interpretation, internalisation, and behavioural adjustment. This pathway is likely to vary across users and contexts, depending on multilingual identity, community support, political awareness, and digital literacy. It may become especially salient when users compare AI performance across languages, when language choice is tied to identity, or when the communicative task has educational, professional, or public consequences. Provisionally, reduced perceived linguistic adequacy may weaken communicative confidence, which may in turn lower willingness to communicate, although this ordering requires empirical testing. The following sections consider each dimension in turn.

### 3.2 Data dominance: unequal linguistic representation and perceived linguistic adequacy

The first layer of algorithmic dominance lies in data dominance. Digital systems do not meet languages on equal terms; they learn from what has been digitised, standardised, annotated, and made computationally useful. The unequal distribution of digital resources is therefore not a secondary technical matter but a basic condition for the reproduction of contemporary language hierarchy. Languages with large corpora, stable orthographies, extensive annotation, and broad domain coverage are far more likely to receive sustained support, whereas languages with sparse data or weaker digital infrastructures remain disadvantaged from the outset (Bender, 2019; Joshi et al., 2020). Here, “low-resource” refers broadly to languages with limited digitised corpora, annotation, benchmarking, and NLP tool support, rather than to speaker numbers alone. What later appears as uneven performance often begins much earlier as uneven representation. For example, Joshi et al. (2020) note that only a very small number of the world’s more than 7,000 languages are represented in rapidly evolving language technologies and applications.

This asymmetry has both psychological and political implications. As Grützner-Zahn et al. (2024) note, language technology support remains deeply uneven across languages, reflecting not only demographic scale but also historical investment, market priorities, and infrastructural privilege. Dominant languages benefit from a cumulative dynamic: the more extensively they are represented, the more effectively they can be processed, and the more likely they are to be used and further digitised. Less-resourced languages, by contrast, are often trapped in a cycle of technical marginality. Their absence from digital corpora may then be misread as evidence of limited relevance rather than structural exclusion. Illustrative examples can be found in both European and Asian contexts. A minor European language such as Welsh, an Asian language such as Lao or Khmer, or a Global South language such as Hausa or Quechua may have institutional recognition, community value, and active everyday use, yet remain less extensively represented than English or other high-resource languages in large-scale corpora, benchmark datasets, and AI-supported services. In such cases, limited AI support should not be interpreted as evidence that these languages are communicatively weak or socially insignificant. Rather, it reflects an infrastructural history in which some languages have been made more machine-readable than others.

For speakers, this matters because data scarcity may shape perceived linguistic adequacy. A language that is weakly represented in digital systems is less likely to feel usable in important domains of online life. Over time, such conditions may foster the sense that one’s language is less suited to knowledge, mobility, or digitally mediated communication. What earlier scholarship identified as a political hierarchy between languages may thus reappear at the level of self-perception.

### 3.3 Platform dominance: visibility, legitimacy, and communicative confidence

If data dominance concerns which languages are available for computation, platform dominance concerns which languages become visible, searchable, and widely circulated in digital environments. Platforms do not merely host communication; they organise attention. Through ranking, recommendation, moderation, interface design, and circulation logics, they help determine which languages travel further, which are more easily encountered, and which come to appear normal in transnational communication (Couldry and Mejias, 2019; Zuboff, 2019). Language hierarchy in the age of digital intelligence is therefore shaped not only by who speaks a language, but by how visibility is distributed through platform infrastructures.

This matters because visibility is closely tied to legitimacy. Languages that already possess strong institutional, commercial, and technological support are better positioned to circulate across platforms, while less dominant languages often remain confined to narrower niches. Zaugg et al. (2022) note that digital marginalisation is reinforced when languages lack both computational support and meaningful presence in the infrastructures through which digital publics are formed. UNESCO (2025) similarly argues that multilingualism in the digital era depends not just on recognition in principle, but on whether languages can function effectively in systems that mediate access and communication. For speakers of Welsh, Lao, or Khmer, using English on social media or professional platforms can sometimes improve searchability, recommendations, and audience reach. Platform dominance, therefore, works not only through exclusion but also by making dominant-language choices appear more publicly viable.

From a psychological perspective, such asymmetry can affect communicative confidence. When some languages are repeatedly presented as more visible, current, or widely usable, speakers may come to see them as safer or more legitimate choices in digitally mediated interaction. Platform dominance shapes not only circulation but also whether language users feel entitled to participate and whether their own repertoire seems publicly viable.

### 3.4 Model dominance: AI performance, language choice, and communicative behaviour

A further dimension of algorithmic dominance is model dominance, that is, the unequal capacity of AI systems to process, generate, and evaluate different languages. This is not reducible to data alone, although data remains foundational. Once trained and deployed, models begin to have effects of their own by rewarding some languages with greater fluency, reliability, and usability than others. In practice, high-resource languages tend to perform better in machine translation, speech recognition, text generation, and related language technologies, whereas less-resourced languages are more likely to be processed unevenly or inaccurately (Joshi et al., 2020; Grützner-Zahn et al., 2024; McGiff and Nikolov, 2025). The result is not merely a technical difference in quality, but a hierarchy of computational ease. Recent large language models (LLMs), including GPT-4o, Claude 3, and DeepSeek, have expanded multilingual access and AI-mediated communication, but broader coverage does not by itself remove deeper performance gaps between high-resource and less-supported languages. For speakers of a less-supported language such as Māori or Irish, weaker speech recognition, machine translation, or generative AI output may make English seem more reliable for study, work, or public communication. Such everyday experiences may gradually shape language choice.

That hierarchy has behavioural and psychological consequences. A language that is translated more accurately, transcribed more reliably, and handled more smoothly by digital systems becomes easier to use in work, education, administration, and everyday interaction. Over time, this functional advantage may feed back into language choice itself. Users may increasingly turn to dominant languages not because they no longer value their own, but because the tools they depend on work better in those languages. Convenience, in other words, may become a quiet mechanism of linguistic displacement.

This is also where the link with psychology becomes especially clear. Differential model performance may shape speakers’ confidence in their own linguistic resources, their willingness to communicate in particular languages, and their expectations of being understood. A language that is repeatedly mishandled by AI systems may come to feel less effective or less worth the effort of public use. By contrast, a language that is seamlessly supported may appear more dependable and more appropriate for high-stakes interaction.

### 3.5 Ideological dominance: from technological advantage to psychological internalisation

The final dimension is ideological dominance, which gives the previous three their wider social durability. Data asymmetry, platform visibility, and model performance do not carry meaning on their own. They become powerful because they are interpreted in ways that conceal their historical and political foundations. This is where language hegemony acquires a particularly durable form in the age of digital intelligence: dominant languages do not simply enjoy greater infrastructural support; that support is easily re-read as proof that they are naturally more efficient, more global, or more suitable for contemporary life (Phillipson, 2022; Zeng et al., 2023). Once this happens, hierarchy no longer appears primarily as hierarchy. It begins to look like common sense.

This internalisation may operate through several related psychological mechanisms. First, repeated technological failure may produce a form of learned constraint: when speakers repeatedly see their language misrecognised, poorly translated, or weakly represented, they may come to anticipate communicative difficulty before interaction even begins. Second, algorithmic asymmetry may create social identity threat, because poor technological support can be experienced not only as a failure of the system, but also as an implicit devaluation of one’s linguistic community. Third, users may misattribute infrastructural limitations to the language itself, interpreting “the system does not handle my language well” as “my language is less suitable for serious digital communication”. In this way, psychological internalisation does not require explicit ideological persuasion. It can emerge gradually through repeated exposure to unequal technological performance, followed by attribution, self-evaluation, and behavioural adjustment.

**Figure 2:**
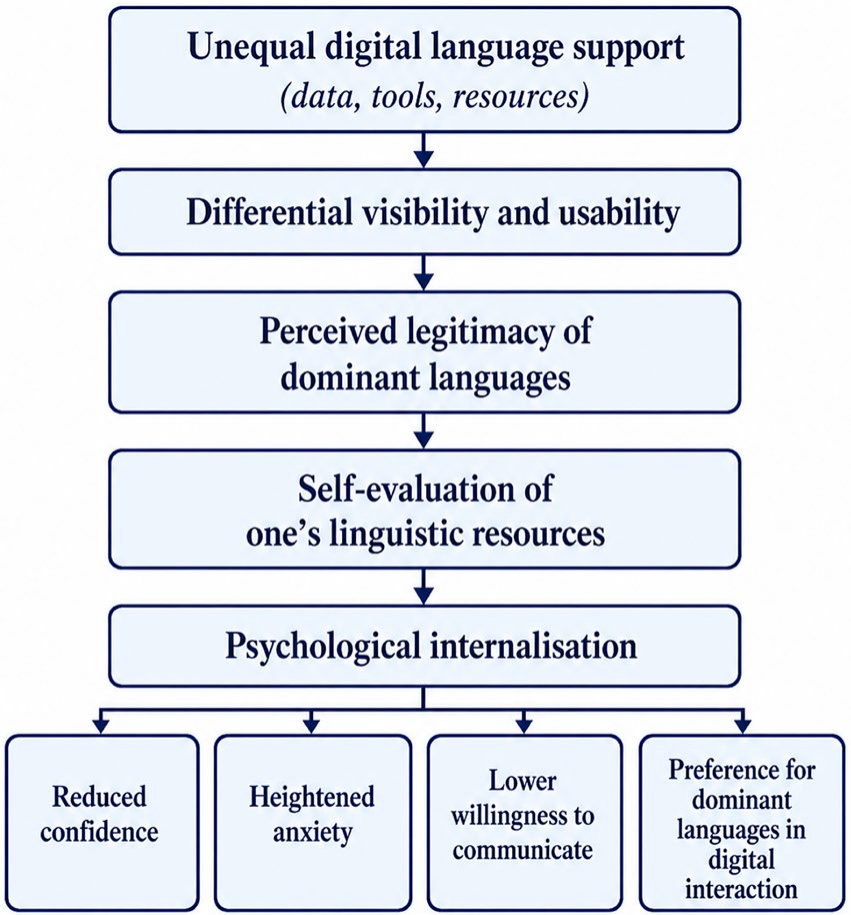
From technological asymmetry to psychological internalisation.

This process matters psychologically because internalisation is part of how hegemony works. A dominant language may come to feel more useful because it is better translated, more visible online, or more smoothly handled by AI systems. Yet these are not innocent reflections of intrinsic merit. They are the cumulative effects of uneven investment, unequal representation, and historically sedimented privilege. As earlier work on linguistic imperialism has shown, domination is most stable when it is naturalised rather than openly imposed (Phillipson, 1992; Holliday, 2006). Under contemporary conditions, that naturalisation increasingly takes technological form. Dominant languages are presented as practical, interoperable, and future-oriented, while less dominant ones are implicitly cast as limited or technically burdensome. Figure 2 illustrates the pathway through which technological asymmetry may be translated into psychological internalisation.

As illustrated in Figure 2, the result is a form of psychological internalisation. Speakers may begin to see dominant languages not only as socially advantageous, but as objectively better suited to expression, participation, or advancement. Conversely, their own languages may come to feel less adequate, less visible, or less worth maintaining in digitally mediated contexts. At that point, language hierarchy is no longer sustained solely by institutions or infrastructure. It is also carried in expectations, anxieties, evaluation habits, and anticipations of communicative success. This process also has an epistemic dimension: when less-supported languages are poorly translated, indexed, or generated, their speakers may be treated as less credible or less visible knowers in digital spaces. This can be understood as a form of epistemic injustice, involving both testimonial and hermeneutical dimensions (Fricker, 2007). Yet such internalisation does not mean that speakers simply accept algorithmic dominance without negotiation. The next section, therefore, considers how language users may resist, adapt to, or strategically work around unequal digital infrastructures.

### 3.6 Resistance, adaptation, and digital code-switching

Algorithmic dominance should not be understood as total or uncontested. Speakers may also resist or adapt through everyday multilingual practices, especially digital code-switching, by moving between dominant and less-supported languages according to audience, platform affordance, emotional attachment, and communicative purpose. Such practices may help users gain visibility in dominant-language spaces while maintaining a sense of local belonging in community-oriented interactions. For example, users may post in English to reach wider publics while using Māori, Irish, Welsh, Lao, Khmer, or other less-supported languages in community spaces to sustain intimacy, identity, and local authority. In this sense, digital code-switching is not merely a pragmatic adjustment, but a way of negotiating visibility and belonging.

Resistance may also take collective forms, such as creating digital content in less-supported languages, contributing to open corpora, or calling for better multilingual interfaces and model evaluation. These practices echo broader accounts of multilingual agency, in which speakers creatively mobilise their linguistic repertoires rather than simply accepting dominant-language norms (Canagarajah, 1999; García, 2009). However, these strategies should not be romanticised, since individual or community-level efforts cannot fully compensate for structural inequalities in data, platform governance, and model development. They may also carry psychological costs, including communicative fatigue, identity tension, and the burden of repeatedly translating oneself for algorithmically dominant spaces.

## 4 Conclusion

This article has argued that language hegemony in the age of digital intelligence is reproduced not only through institutions, ideology, and historical prestige, but also through computational infrastructures that differentially support, circulate, and normalise languages. For that reason, algorithmic dominance is proposed not as a replacement for linguistic imperialism, but as a way of extending it into the present. What is at stake is not simply which languages are more visible or more usable online, but how such conditions shape speakers’ sense of legitimacy, confidence, communicative possibility, and responses of adaptation or resistance.

From a psychological point of view, these asymmetries matter because they are likely to enter everyday experience. A language that is poorly supported in digital systems may come to feel less adequate for public use, while one that is seamlessly processed and widely visible may appear more reliable, more legitimate, and less risky to use. In this way, the language hierarchy is not only sustained externally; it may also be internalised as lowered confidence, heightened anxiety, or reduced willingness to communicate.

This article is conceptual and does not test these claims empirically. Rather than presenting empirically verified hypotheses, it offers a theoretical framework and a set of conceptual propositions for future research. That limitation also points to the next step. Future research should examine more directly how unequal digital language support affects speakers’ self-perception, communicative confidence, language anxiety, willingness to communicate, language choice, and digital code-switching across different sociolinguistic settings.

If language inequality is increasingly reproduced through digital systems, then multilingual justice can no longer be treated solely as a matter of policy or education. It is also a matter of how languages are designed into the infrastructures through which people now read, write, speak, and are heard.

## Data Availability

The original contributions presented in the study are included in the article/supplementary material, further inquiries can be directed to the corresponding author.
